# Genome sequence and rapid evolution of the rice pathogen *Xanthomonas oryzae *pv. oryzae PXO99^A^

**DOI:** 10.1186/1471-2164-9-204

**Published:** 2008-05-01

**Authors:** Steven L Salzberg, Daniel D Sommer, Michael C Schatz, Adam M Phillippy, Pablo D Rabinowicz, Seiji Tsuge, Ayako Furutani, Hirokazu Ochiai, Arthur L Delcher, David Kelley, Ramana Madupu, Daniela Puiu, Diana Radune, Martin Shumway, Cole Trapnell, Gudlur Aparna, Gopaljee Jha, Alok Pandey, Prabhu B Patil, Hiromichi Ishihara, Damien F Meyer, Boris Szurek, Valerie Verdier, Ralf Koebnik, J Maxwell Dow, Robert P Ryan, Hisae Hirata, Shinji Tsuyumu, Sang Won Lee, Pamela C Ronald, Ramesh V Sonti, Marie-Anne Van Sluys, Jan E Leach, Frank F White, Adam J Bogdanove

**Affiliations:** 1 Center for Bioinformatics and Computational Biology, University of Maryland, College Park, MD 20742, USA; 2 The Institute for Genomic Research, Rockville, MD 20850, USA; 3 Institute for Genome Sciences, University of Maryland, Baltimore, MD 21201, USA; 4 Laboratory of Plant Pathology, Kyoto Prefectural University, Sakyo, Kyoto 606-8522, Japan; 5 Department of Genetic Resources, National Institute of Agrobiological Sciences, Kannondai, Tsukuba 305-8602, Japan; 6 Current address: J. Craig Venter Institute, Rockville, MD 20850, USA; 7 Current address: National Center for Biotechnology Information, National Institutes of Health, Bethesda, MD 20894, USA; 8 Centre for Cellular and Molecular Biology, Council of Scientific and Industrial Research, Hyderabad, India; 9 Institute of Himalayan Bioresource Technology, Council of Scientific and Industrial Research, Palampur, India; 10 Department of Bioagricultural Sciences and Pest Management, Colorado State University, Fort Collins, CO, USA; 11 Department of Plant Pathology, Iowa State University, Ames, IA, USA; 12 Institut de la Recherche pour le Developpement, 911 Av. Agropolis, Montpellier, 34090, France; 13 BIOMERIT Research Centre, BioSciences Institute, University College Cork, Cork, Ireland; 14 Graduate School of Natural Science & Technology, Shizuoka University, 836 Ohya, Suruga-ku, Shizuoka, 422-8017, Japan; 15 Department of Plant Pathology, UC Davis, Davis, CA 95616, USA; 16 Departamento de Botânica, IB-USP, Sao Paulo, SP, Brazil; 17 Department of Plant Pathology, Kansas State University, Manhattan, KS, USA

## Abstract

**Background:**

*Xanthomonas oryzae *pv. oryzae causes bacterial blight of rice (*Oryza sativa *L.), a major disease that constrains production of this staple crop in many parts of the world. We report here on the complete genome sequence of strain PXO99^A ^and its comparison to two previously sequenced strains, KACC10331 and MAFF311018, which are highly similar to one another.

**Results:**

The PXO99^A ^genome is a single circular chromosome of 5,240,075 bp, considerably longer than the genomes of the other strains (4,941,439 bp and 4,940,217 bp, respectively), and it contains 5083 protein-coding genes, including 87 not found in KACC10331 or MAFF311018. PXO99^A ^contains a greater number of virulence-associated transcription activator-like effector genes and has at least ten major chromosomal rearrangements relative to KACC10331 and MAFF311018. PXO99^A ^contains numerous copies of diverse insertion sequence elements, members of which are associated with 7 out of 10 of the major rearrangements. A rapidly-evolving CRISPR (clustered regularly interspersed short palindromic repeats) region contains evidence of dozens of phage infections unique to the PXO99^A ^lineage. PXO99^A ^also contains a unique, near-perfect tandem repeat of 212 kilobases close to the replication terminus.

**Conclusion:**

Our results provide striking evidence of genome plasticity and rapid evolution within *Xanthomonas oryzae *pv. oryzae. The comparisons point to sources of genomic variation and candidates for strain-specific adaptations of this pathogen that help to explain the extraordinary diversity of *Xanthomonas oryzae *pv. oryzae genotypes and races that have been isolated from around the world.

## Background

*Xanthomonas oryzae *pathovar oryzae (Xoo), a member of the gamma subdivision of the proteobacteria, is a major pathogen of rice (*Oryza sativa *L.). It enters rice leaves through water pores or wounds and moves systemically by invading the xylem, causing a disease known as bacterial blight [1]. Bacterial blight is the most serious bacterial disease of rice, and in some areas, the most important of any disease of rice, carrying the potential to reduce yields by as much as 50% [2]. When Xoo infects at the seedling stage, it causes a syndrome known as kresek, which can lead to nearly complete crop loss [1]. Several factors that contribute to fitness and virulence in Xoo have been identified (reviewed in [3]). However, as rice is a staple crop for much of the world population, as well as a model for cereal biology [4], a better understanding of pathogenesis by Xoo remains a pressing goal both for control of bacterial blight and for fundamental understanding of bacterial-plant interactions.

Bacterial blight occurs in most rice growing areas of the world, and Xoo isolates from within and across Africa, India, Asia, and Australia show a great diversity of genotypes, based on polymorphism of transposable elements, predominantly insertion sequences (IS), avirulence genes, rep/box elements, and other markers [5]. Based on the ability of strains to elicit resistance in particular host genotypes, several distinct races have been defined [2]. Rice is one of our most ancient domesticated crops, and comprises more than 100,000 distinct varieties [6]. Twenty nine bacterial blight resistance (*R*) genes (*Xa1-Xa29*) have been identified to date [7]. The great diversity of strains within Xoo undoubtedly reflects adaptation of the pathogen to the diversity of host genotypes as well as the diverse environmental conditions in which rice is grown. From a broader perspective, Xoo belongs to a diverse and highly adapted genus that includes more than 20 plant-associated or plant pathogenic species. Each species may comprise one or more pathogenic varieties (pathovar; pv.), which demonstrate distinct host plant specificity or modes of infection. Collectively, different *Xanthomonas *species and pathovars cause diseases in over 390 host plant species [8].

Complete genome sequences have been published for two strains of Xoo, MAFF311018 (MAFF), a Japanese race 1 strain also referred to as T7174 [9], and KACC10331 (KACC), a Korean race 1 isolate also known as KXO85 [10]. Comparative analysis of multiple Xoo genomes promises insight into specific adaptations that allow different strains to maintain virulence in different types of rice in different regions of the world. Of particular potential interest are adaptations involving extracellular components, and type III effectors, which have been established as critical virulence factors in bacterial blight or other plant bacterial diseases [3,11].

The genomes of MAFF and KACC overall are highly similar to one another in gene content and organization. We report here the complete genome sequence of a third strain of Xoo, PXO99^A^, which, as described below, is considerably more distant from either of these strains than they are from each other. PXO99^A ^is a 5-azacytidine-resistant derivative of PXO99, which was isolated in Los Baños and classified as Philippine race 6 [12]. Genotypically, however, PXO99 is more similar to isolates from South Asia (Nepal and India) than to other Philippine isolates [13]. In contrast to MAFF and KACC, PXO99^A ^is virulent toward a large number of rice varieties representing diverse genetic sources of resistance, including the broad-spectrum, recessive resistance gene *xa5 *[14]. The relatively few resistance genes effective against PXO99^A ^include the recessive resistance gene *xa13*, which is ineffective against MAFF and KACC, the recently characterized broad-spectrum resistance gene *Xa27*, and the pattern recognition receptor-like resistance gene *Xa21*, which is effective against MAFF but not against KACC [15-17]. Because of its amenability to genetic analysis, and its relatively broad cultivar specificity, PXO99^A ^has been the focus of numerous studies of the molecular basis of bacterial blight and blight resistance.

## Results

### The PXO99^A ^genome

**Figure 1:**
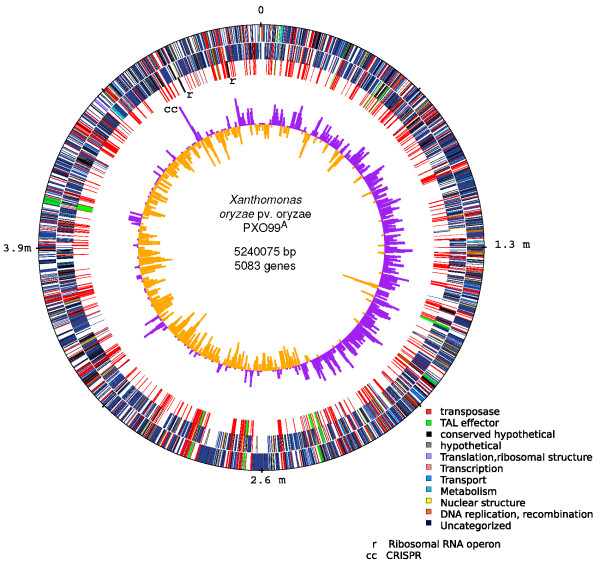
**Circular representation of the *Xanthomonas oryzae *pv. oryzae genome**. Rings illustrate, from outside to inside: protein coding genes (forward strand), protein coding genes (reverse strand), TAL effectors (green) and IS elements (red), and GC-skew plot showing (G-C)/(G+C) in 10 kilobase windows. Positive values of GC-skew indicate the leading strand of replication, negative values the lagging strand.

The PXO99^A ^genome is a single circular chromosome of 5,240,075 bp with an overall GC content of 63.6%. It contains 5083 protein-coding genes, 2 ribosomal RNA operons, and 55 tRNAs (Table 1). The origin of replication was identified by similarity to other *Xanthomonas *genomes, by proximity to genes (*dnaA*, *dnaN*, and *gyrB*) often found near the origin on bacterial genomes, and by GC-skew analysis, which examines the excess of G versus C on the leading strand [18]. A schematic representation of the genome is provided in Figure 1.

**Table 1:** Comparison of 3 *Xanthomonas oryzae *pv. oryzae genomes

	PXO99^A^	KACC	MAFF
Length (bp)	5,240,075	4,941,439	4,940,217
GC content (%)	63.6	63.7	63.7
Annotated genes	5,083	4,637	4,372
IS elements (complete/fragment)	267 (683)	252 (714)	251 (712)
TAL effector genes	19	15	17

### Relationship to other sequenced *Xanthomonas oryzae *genomes

To assess the phylogenetic relationships among PXO99^A ^and related strains, we aligned the complete genome to the genomes of MAFF, and KACC, and strain BLS256 of *X. oryzae *pv. oryzicola, (GenBank Accession AAQN01000001), and generated a cladogram using Mauve 2.1.1 [19]. MAFF and KACC group together, but PXO99^A ^is clearly distinct and considerably more distant from MAFF and KACC than they are from one another (Additional file 1). The tree was confirmed by another tree built with all the sequenced *Xanthomonas *genomes and rooted with *Xylella fastidiosa *(Temecula strain) (data not shown).

### Genes unique to PXO99^A ^relative to MAFF

Of the 5083 annotated protein coding genes in PXO99^A^, 4910 have clear homologs in the MAFF strain. These genes map to just 4234 genes in MAFF (out of 4372 total), indicating a considerable expansion of some gene families. 194 of the shared genes are present in a 212 kb direct repeat near the replication terminus (see below). Of the remaining 173 PXO99-specific genes, 29 (including 18 tranposases) are missing from MAFF because they span breakpoints; i.e., a rearrangement, insertion, or deletion in MAFF has broken these genes into fragments. Fifty eight other PXO99^A ^genes only partially align to MAFF, including 29 transposases. Finally, 86 genes in PXO99^A ^are completely absent (based on sequence alignment) from MAFF.

Among the 138 annotated genes in the MAFF strain that are not present in PXO99^A^, 20 are missing in PXO99^A ^because they span breakpoints, and 38 (including 12 transposases) are missing because they are truncated in PXO99^A^. The remaining 80 genes in MAFF are entirely missing from PXO99^A^.

Additional file 2 contains the lists of genes unique to PXO99^A ^and unique to MAFF. It is noteworthy that a majority of the genes unique to MAFF (64/80) are hypothetical proteins, which may represent annotation artifacts. These hypothetical genes have an average length of 182 bp, compared to 850 bp for an average gene. Of the 87 genes unique to PXO99^A^, twenty are hypothetical while the remainder comprises genes similar to predicted genes in other strains and species.

### IS elements

All sequenced *Xanthomonas *genomes contain numerous IS elements, but the Xoo genomes contain the most diverse pool [20]. Of the 19 known families of IS elements [21], eight families composed of 28 distinct elements appear in Xoo. MAFF and KACC have nearly identical numbers of IS elements (Table 1), while PXO99^A ^contains fewer elements overall, but more copies of ISXo8, IS1114/ISXoo4, and ISXo2.

### A genomic region encoding several non-fimbrial adhesin genes

**Figure 2:**
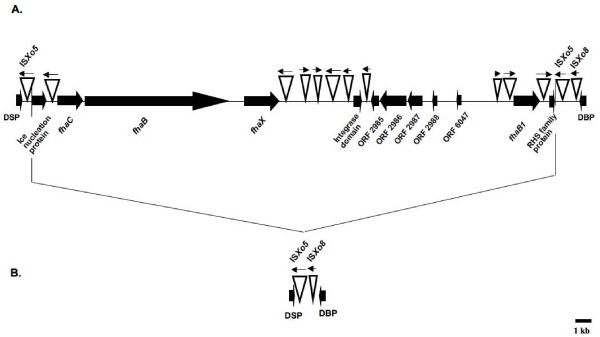
**A 38.8 kb region including nonfimbrial adhesin genes that is unique to PXO99^A^**. A: organization of the region in the PXO99^A ^genome. Block arrows represent genes; inverted triangles represent insertion sequence elements. The region is flanked by DSP (dual specificity protein) and DBP (DNA binding protein) encoding genes, which are also present in MAFF and KACC. B: the corresponding locus in MAFF and KACC, missing the entire block of genes. The point of insertion/deletion maps to an ISXo5 insertion sequence element between DSP and DBP.

Sequences unique to PXO99^A ^relative to MAFF include a 38,766 bp region (coordinates 4788763 – 4827529) that contains several predicted non-fimbrial adhesin genes (Figure 2). Of 20 genes at this locus, three (*fhaB*, *fhaX *and *fhaB1*) encode non-fimbrial adhesin related proteins and a fourth (*fhaC*) is predicted to help in transport of non-fimbrial adhesins. The *fhaB *gene, which encodes the longest protein (3527 aa) in PXO99^A^, contains a hemagglutination activity domain and filamentous hemagglutinin repeats that are likely to serve in adhesion and autoaggregation. Two more genes (ORFs 2986 and 2987) are predicted to be involved in bacteriocin secretion while another (ORF 2973) encodes an ice-nucleation protein homolog. The locus also includes several IS elements and is flanked by direct repeats of ISXo5. These are in turn flanked by genes for a dual specificity phosphatase (DSP in Figure 2) and a DNA binding protein (DBP). In contrast, only one copy of the ISXo5 element is present between DSP and DBP in MAFF and KACC, indicating that the IsXo5 element was involved in the genomic rearrangement that led either to loss of the locus from MAFF and KACC or gain of the locus in PXO99^A^. The former is likely the case because the arrangement in PXO99^A ^is present also in *X. oryzae *pv. oryzicola BLS256 (data not shown).

Specific primers were developed for the DSP and DBP genes that flank this locus as well as for the *fhaB*, *fhaC*, and *fhaX *genes. Using PXO99^A ^genomic DNA as a template, we amplified the expected PCR products for all five genes (data not shown). Using either MAFF 311018 or KACC 10331 genomic DNA as template, products of the expected size were obtained with primers specific to the DBP and DSP encoding genes, but no products were obtained with primers specific for *fhaC*, *fhaB *or *fhaX*. Also, a fragment of the expected size (~2.5 kb) was obtained via PCR with DBP- and DSP-specific primers using MAFF and KACC genomic DNA, but not with PXO99^A ^genomic DNA (data not shown). These results provide additional evidence that the non-fimbrial adhesin genes are indeed missing from the MAFF and KACC genomes. Based on PCR analysis using the above primers, the *fhaC*, *fhaB *and *fhaX *genes are also missing from the Indian Xoo strain BXO43, and in another Indian strain, BXO8, only *fhaB *appears to be present. However, all three genes were detected in the strain Nepal624 (data not shown), a result consistent with the close relationship, as established by DNA fingerprinting studies, between PXO99^A ^and Xoo strains from Nepal [22].

### Recent large duplication

**Figure 5:**
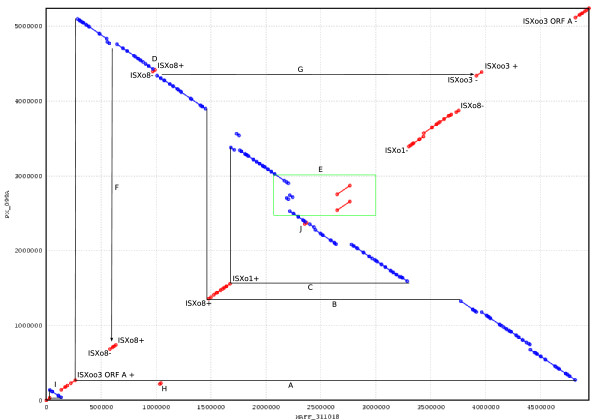
**Inversions and rearrangements in PXO99^A ^compared to MAFF**. The alignment shows regions of PXO99^A ^that align to the same (red) or opposite (blue) strand of MAFF. Transposase genes and their orientation (+ or -) are shown at the sites of each rearrangement. Letters A-J indicate specific rearrangement events. A: the IS element ISXoo3 is composed of two distinct and independently conserved ORFs and is responsible for an inversion spanning coordinates 267869–5114959 (all coordinates refer to the PXO99^A ^genome). B: ISXo8 occurs in opposite orientation at each end of a 2.6 Mbp inversion spanning positions 1356757–3898472. C: ISXo1 occurs in inverted copies at the endpoints of a 1.8 Mbp inversion spanning 1558996–3391786. D: a 33270 bp inverted region spanning 4394742–4428012 is flanked by oppositely-oriented copies of ISXo8. E: Each copy of the 212-kb duplication is flanked by ISXo5, which also occurs adjacent to two other translocations in this region. The duplication appears as two parallel diagonal lines in this box. F: ISXo8 also occurs in inverted copies at the boundaries of a 47540 bp segment that is translocated from approximately 4800000 to 685272. G: ISXoo3 flanks both ends of a 47540 bp translocation from approximately 1117000 to 4339239. H: A 9,862 bp region occurs in inverted copies at 217,455 and 4,305,307. MAFF311018 contains only one copy of this region. I,J: Segments spanning 96,753 bp (I) and 17,021 bp (J) are inverted with respect to MAFF311018 but not associated with transposases.

The PXO99^A ^strain contains a near-perfect tandem duplication of 212,087 bp. This unusually large repeat spans the intervals 2,502,622–2,714,708 and 2,714,709–2,926,795. The repeat is flanked by an insertion (1073 bp) of ISXo5 (Figure 5) at each end and between the two copies. Except for a single base difference in one IS copy, the two regions are 100% identical. Because the flanking ISXo5 is longer than a read, and because the repeat is much too long to be spanned by any pair of sequencing reads, the original assembly had collapsed these two repeats into a single region. Also, the positioning of the flanking short repeats meant that every sequence fit accurately into the collapsed assembly, with only the paired-end information indicating a problem. This collapse was discovered through the use of the Hawkeye assembly diagnostics tool, which identified a large set of mis-oriented paired-end sequences on either end of the collapsed version of the assembly [23]. In order to provide additional validation of this duplication, we designed primers on either side of the unique junction where the two copies of the tandem repeat meet (see Additional file 2). We verified the presence of the junction by PCR amplification and re-sequencing of this region.

The 212 kb segment occurs once in the MAFF and KACC sequences. One question is whether the difficulty of assembling this region might mean that it is present in these strains, but undetected. Evidence that the duplication is indeed unique to PXO99^A ^is the sequence divergence (~0.3%) of PXO99^A ^from MAFF/KACC. This divergence implies that if the duplication had happened in a common ancestor, then the two distinct 212 kb regions, which would have existed since the divergence between strains, would be expected to have over 600 single-base differences. The fact that the copies have only one difference confirms that the large duplication in PXO99^A ^occurred much more recently than its divergence from MAFF and KACC.

### TAL effector genes

**Figure 3:**
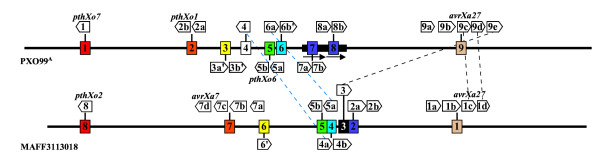
**Relationship of TAL effector genes in Xoo strains PXO99^A ^and MAFF**. The individual genes, distributed among nine loci in PXO99^A ^and eight in MAFF, are represented by open arrows and labeled as described in the text. Pseudogenes (truncated genes or genes with early stop codons) are indicated by an apostrophe. Genes that have identical repeat regions based on number of repeats and identity at the twelfth and thirteenth codons are connected with a black dashed line. Blue dashed lines connect genes with nearly identical repeat regions (see text). Names of previously characterized genes are centered above or below the corresponding open arrow. Colored boxes indicate TAL gene clusters (not to scale), with the same color representing loci at the same relative positions in the two genomes. Locus 4 in PXO99^A ^and locus 3 in MAFF are uniquely positioned in their respective genomes. The solid black rectangle and arrows beneath it represent the 212 kb direct repeat in the PXO99^A ^genome.

A hallmark of the Xoo genome is the large number of transcription activator-like (TAL) type III effector genes, which are defined by their relatedness to the type members *avrBs3 *and *pthA *[24-26]. TAL effector genes are characterized in part by a region of 102 bp repeats, or more rarely 105 bp repeats, within the central coding portion [27,28]. Nineteen TAL effector genes were identified in the PXO99^A ^genome (Table 2 and Figure 3), including four previously associated with virulence and avirulence phenotypes and effector-specific gene expression in rice [16,29,30]. One of these, *pthXo1*, encodes the major virulence determinant for PXO99^A ^whose function is disrupted in rice by the recessive blight resistance gene *xa13 *[29,30]. The TAL effector genes are located in nine loci distributed in the genome. Two loci consist of single genes, six consist of two genes oriented in the same direction, and one is a previously identified cluster of five genes all oriented in the same direction [16]. Each of the genes within a cluster is preceded by a region of 990 bp that contains two or more short, predicted ORFs but is more likely non-coding DNA, suggesting that each gene has its own promoter, and that the clusters do not represent polycistronic operons. We have designated the genes numerically according to the locus in which they reside, sequentially from the origin of replication, and alphabetically, according to their position in that locus starting at the 5' end of the locus. Thus, the first TAL effector gene in the genome sequence, proximal to the origin, is *tal1*, the second (which is the second gene in a locus oriented toward the origin) *tal2b*, *etc*. The genes with known phenotypes are distributed in separate loci: *tal1 *is *pthXo7*, *tal2b *is *pthXo1*, *tal5b *is *pthXo6*, and *tal9c *is *avrXa27*. Among the genes, the number of repeat units varies from 12.5 (*tal9d*) to 26.5 (*tal9c*). None of the genes contains the rare 105 bp repeat. Gene pairs in loci 7 and 8 are identical copies in the 212 kb duplicated regions of the genome.

**Table 2:** TAL effector genes in PXO99^A^

**Gene**	**ID**	**Coordinates**	**Strand**	**Repeats**	**Comments^1^**
*pthXo7 (tal1)*	03922	559109..562222	-	21.5	*OsTFIIAγ1*
*pthXo1 (tal2b)*	00227	1645240..1649043	-	23.5	*Os8N3*
*tal2a*	00223	1650351..1653557	-	14.5	
*tal3a*	00511	1860212..1862083	+	17.5	N-term deletion, truncated
*tal3b*	00505	1864934..1866895	+	17.5	N-term deletion, truncated
*tal4*	00318	2083533..2085968	-	15.5	
*pthXo6, (tal5b)*	00572	2354996..2358139	-	22.5	*OsTFX1*
*tal5a*	00567	2360008..2362440	-	15.5	
*tal6a*	00546	2384284..2387193	+	19.5	
*tal6b*	05609	2388988..2392041	+	20.5	N-term frameshift
*tal7a*	05633	2683629..2686343	+	17.5	
*tal7b*	01085	2688137..2691088	+	19.5	
*tal8a*	06229	2895716..2898430	+	17.5	Duplicate of *tal7a*
*tal8b*	06234	2900224..2903175	+	19.5	Duplicate of *tal7b*
*tal9a*	02172	4101543..4104803	+	19.5	
*tal9b*	05714	4106597..4110244	+	26.5	
*avrXa27, (tal9c)*	05718	4112038..4114644	+	16.5	*Xa27*
*tal9d*	02269	4116438..4118642	+	12.5	
*tal9e*	02272	4120436..4123759	+	23.5	

^1^The rice gene induced by the effector is in italics.

**Figure 4:**
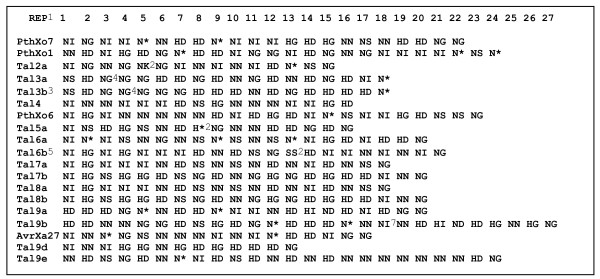
**Alignment of PXO99^A ^TAL effector repetitive regions as represented by the twelfth and thirteenth residues of each repeat**. Notes: 1 * indicates a proposed deletion of the thirteenth codon in the repeat; 2, novel variable codons; 3, truncation; 4, six-codon deletion; 5, N-terminal frameshift; 6, five-codon deletion in repeat.

With the exception of the gene pairs within the 212 kb duplication, none of the genes share the same repeat region structure based on a comparison of the twelfth and thirteenth codons, which vary from repeat to repeat (Figure 4). Genes *tal3a *and *tal3b *each have two deletions of 43 and 15 codons in their 5' ends and are truncated in the 3' ends of their coding regions, so they are unlikely to produce functional effectors. The similarities in *tal3a *and *tal3b *indicate that one is a duplicate of the other. Gene *tal6b *has a frameshift mutation within the 5'-end of the coding region and is therefore also unlikely to be functional. *Genes tal6b*, *tal7b*, and *tal8b *share a novel eleven codon duplication (PERTSHRVADL-PERTSNRVADL) at their 3' ends.

Comparison of the TAL effector gene content and arrangement in PXO99^A ^with those in MAFF, using the scheme described above to name the MAFF genes (genes in the two strains are hereafter distinguished by subscript), indicates that the number of loci and relative positions are similar with the exception of the duplicated loci (7 and 8) in PXO99^A^, as well as PXO99^A ^locus 4 and MAFF locus 3, which occupy unique relative positions in their respective genomes (Figure 3). MAFF loci 2 and 3 were considered by Ochiai et al. [9] as one locus, but we treat them as distinct based on the unusual distance (roughly 3 kb instead of the usual 990 bp) between the locus 3 gene and the closest locus 2 gene, and the presence of IS elements flanking locus 3. Despite the similarity in number and arrangement of the respective loci, only three PXO99^A ^TAL effector genes, all in PXO99^A ^locus 9, have counterparts in MAFF that are identical with respect to the number of repeats and the twelfth and thirteenth codons of the central repeat domain. The identical genes are *tal9c*_PXO99A _and *tal1c*_MAFF_, *tal9d*_PXO99A _and *tal1d*_MAFF_, and *tal9e*_PXO99A _and *tal3*_MAFF_. Genes *tal9a*_PXO99A _and *tal9b*_PXO99A _correspond in repeat number to *tal1a*_MAFF _and *tal1b*_MAFF_, respectively. The *tal3*_MAFF _gene, which represents a break in the apparent overall synteny between PXO99^A ^locus 9 and MAFF locus 1, is flanked by IS elements. The *tal9c*_PXO99A _gene is the avirulence determinant *avrXa27*, and its identity with *tal1c*_MAFF _is consistent with the effectiveness of the corresponding host resistance gene *Xa27 *against both PXO99^A ^and MAFF, as well as a broad range of other strains [31]. Two other PXO99^A ^TAL effector genes have counterparts in MAFF that are nearly identical with respect to the number of repeats and the predicted twelfth and thirteenth residues in each repeat: *tal4*_PXO99A _has the same structure as *tal4a*_MAFF _except for residue 12 in the fifteenth repeat, and *tal6a*_PXO99A _has the same structure as *tal5a*_MAFF _except for residues 12 and 13 in the fourteenth repeat. MAFF has two TAL effector genes, *pthXo2 *(*tal8*_MAFF_) and *avrXa7 *(*tal7d*_MAFF_), that are major virulence determinants [30]. The *pthXo2 *gene occupies the same locus in MAFF that *pthXo7 *does in PXO99^A^, while *avrXa7 *occupies the same locus as *pthXo1*, the major virulence determinant for PXO99^A^. Some corresponding loci differ in their gene content. For example, locus 2 in PXO99^A ^consists of two genes but the corresponding locus in MAFF, locus 7, contains four. Absent from MAFF locus 5 is *pthXo6*, although previous evidence indicates that *OsTFX1*, a host gene expressed in a *pthXo6*-dependent manner, is induced upon infection with MAFF [32]. Induction could be due to one of the other TAL effectors, or *pthXo6 *might have been misassembled in the MAFF sequence. Locus 6 in PXO99^A ^corresponds to locus 4 in MAFF, but locus 4 in MAFF contains the gene nearly identical to *tal4*_PXO99A _in the uniquely positioned locus 4 of PXO99^A^. The MAFF gene nearly identical to *tal6a*_PXO99A_, is located in a corresponding neighboring locus, MAFF locus 5. Locus 3 in PXO99^A ^and 6 in MAFF contain two and one defective TAL effector genes, respectively. All three of these genes have identical repeat domains. Moreover, *tal6*_MAFF _shares with the PXO99^A ^genes the 3' deletions of 43 and 15 codons discussed above, as well as a six-codon deletion in the repeat region (repeat 4 of *tal6*_MAFF_, repeat 3 of *tal3a*_PXO99A_, and repeat 4 of *tal3b*_PXO99A_), indicating that these genes may represent a generally defunct locus in Xoo. The observed substitution of genes at conserved loci across the genomes, expansion or contraction of individual loci in a given strain, and divergence or degeneration of gene sequences at shared loci are presumably accomplished by the exchange of coding sequences through homologous recombination. Transposition of genes involving IS element-mediated recombination may also occur, as exemplified possibly by *tal3*_MAFF_.

### Genome rearrangements in Xoo

The PXO99^A ^strain of Xoo has experienced at least ten major rearrangements with respect to the MAFF strain, resulting in 29 distinct syntenic blocks, as shown in Figure 5. The majority of these rearrangements are symmetric about the origin of replication, as has been observed for many other bacterial rearrangements [33]. Most of these rearrangements appear to be mediated by a diverse set of transposable elements. Some elements, such as ISXo5, ISXo8, and IS1389/ISXoo3, are responsible for multiple rearrangements. For example, ISXo5 occurs near each endpoint of both copies of the 212,087 bp tandem repeat (region E, Figure 5). Within each copy of the repeat there is a 116,872 bp inversion flanked by inverted copies of ISXo5. Only three major rearrangement events (H, I, and J in Figure 5) do not seem to be associated with IS elements.

### Evolution of the CRISPR region in Xoo lineages

The PXO99^A^, MAFF, and KACC genomes each contain a CRISPR (clustered regularly interspersed short palindromic repeats) element. CRISPRs are identified by a set of Cas genes, followed by a leader sequence and then a variable number of alternating spacers and repeats; the elements here represent the Dvulg subtype [34]. The repeats are identical, while the spacers represent foreign DNA that was laterally transferred from a bacteriophage or a plasmid [35]. A growing body of evidence demonstrates that the spacers, acquired during phage infection, provide immune protection for the bacterium against the phage [36]. Thus CRISPRs represent an inheritable immune system for bacteria.

**Figure 6:**
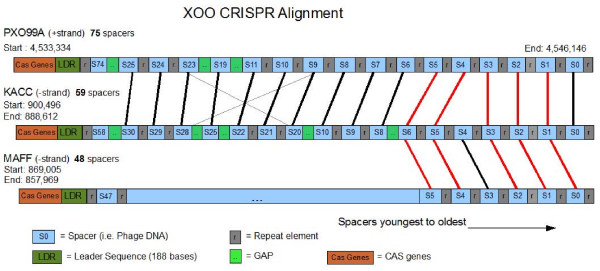
**Alignment of CRISPR elements from the PXO99^A^, KACC, and MAFF genomes**. Spacers are numbered from right (S0) to left, with the oldest elements on the right. Gaps (green boxes) indicate the positions of additional spacers in the genomes not shown here. Red lines indicate spacers shared in all three genomes, heavy black lines indicate spacers shared in just two species, and thin black lines indicate spacers that are similar but not identical between two species.

Because the CRISPR region evolves very rapidly, it provides one of the most striking records of differentiation among PXO99^A^, MAFF, and KACC. As shown in Figure 6, PXO99^A ^has the largest CRISPR region of the three strains, with 75 spacer elements. In contrast, MAFF and KACC contain just 48 and 59 spacers respectively, implying that PXO99^A ^has acquired a substantially greater resistance to phage infections than its cousins. Also worth noting is that the majority of the spacers are unique to each strain, attesting to the rapid evolution of these regions.

The alignment of the CRISPR spacers in the three Xoo strains (Figure 6) appears on first inspection to contradict the phylogenetic relationship of the strains, in that MAFF appears more distant from the other two strains. Spacers are inserted into a genome in chronological order, with new elements appearing next to the 188-bp leader sequence, which gives a clear picture of the shared history of these elements. Our alignment shows that all three strains share five of the oldest elements (S1–S5 in PXO99^A^), but that all of the more recent elements in MAFF are unique to that strain. PXO99^A ^and KACC share the very oldest element, which has been lost in MAFF, as well as 10 additional older spacer elements in conserved order. These 10 spacers range from S6–S25 in PXO99^A ^and S8–S30 in MAFF (intervening elements are unique in each strain), indicating that these two strains diverged after the acquisition of spacer S25/S30. MAFF, in contrast, shares no spacers more recent than S5 with either of the other two strains. This appears to contradict whole-genome phylogenetic evidence and large-scale genome structure, both of which indicate that MAFF and KACC are much closer to one another than either is to PXO99^A^. A likely alternative explanation, given the hypervariable nature of CRISPRs, is that MAFF lost these older spacers.

### Validation of the MAFF assembly

To validate the large-scale rearrangements between strains PXO99^A ^and MAFF, we obtained a library of 9 kb shotgun clones for MAFF and identified those clones that correspond to breakpoints shown in Figure 5. Two clones for each breakpoint were selected, except in one case where only one clone could be identified. These clones were end-sequenced and the ends compared to the MAFF genome. In addition, restriction enzyme analysis was performed for each of the shotgun clones.

In all cases, the analysis of the MAFF sequences confirmed that the MAFF genome is correctly assembled. Had there been any mis-assemblies, the clones would have shown significant length polymorphisms or would have mapped to inconsistent positions on the finished sequence. This evidence further strengthens the conclusion that breakpoints in the genome alignment between MAFF and PXO99^A ^represent genuine differences between the genomes. Because the MAFF and KACC strains have almost the same overall genome architecture, with very few rearrangements, we did not attempt separate verification of the KACC assembly.

Separately, we identified 18 significant insertions and deletions between MAFF and PXO99^A^. We generated PCR primers to test for the presence or absence of each insertion, and amplified fragments from genomic DNA using both strains. In all cases the PCR tests verified the presence of the insertion in one strain and its absence in the other (data not shown).

### Regions of lateral gene transfer

**Figure 7:**
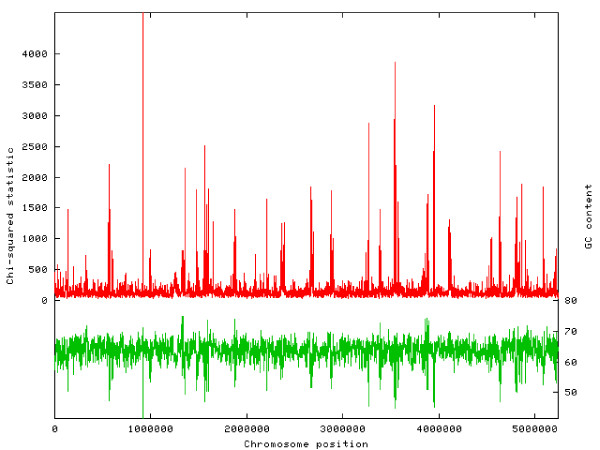
**Compositional analysis of the PXO99A genome**. Analysis of genome composition in 1000 bp windows. The red plot shows a X^2 ^analysis, in which the trinucleotide composition of each window is compared to the overall composition. The green plot shows GC content for the same windows.

GC-content frequently used for identifying regions of a genome with unusual composition, as might result from lateral gene transfer. PXO99^A ^has a GC-content of 63.6%, ranging from a high of 71.8% to a low of 41.6%. A more sensitive measure of unusual composition, used in many previous studies (e.g., [37]) is based on trinucleotide composition. For this measure, we compute the X^2 ^statistic to compare the trinucleotide distribution in fixed-size windows to the overall trinucleotide distribution for the genome. Regions highlighted by this statistic are either caused by lateral gene transfer or else under very strong evolutionary constraints to maintain their atypical DNA composition. A plot of the X^2 ^statistic as well as GC-content across the genome is shown in Figure 7.

The figure shows multiple regions of highly unusual composition, which we then investigated further. The largest peak in the X^2 ^distribution, at position 918,000, is centered on a 424-aa protein (ORF04252) containing a lysin domain (often found in enzymes involved in bacterial cell wall degradation) but whose function is otherwise unknown. There is strong evidence that this gene has been laterally transferred via a bacteriophage: it is not found in any other Xanthomonads, and the closest matches are in Burkholderia, Campylobacter, and Shewanella, all very distantly related genera. Homologs in both *B. pseudomallei *K96243 [38] and *Erythobacter litoralis *are annotated as acquired from bacteriophage, and a direct phage homolog occurs in Burkholderia phage phiE202. A phylogenetic tree of all homologs (data not shown) supports the conclusion that this gene was laterally transferred via a phage.

The second-highest peak in Figure 7 is in the midst of a broader region of unusual composition, extending from 3,540,900 to 3,571,800. This region contains a large prophage element with 41 phage-related genes, extending from ORF01364 (a phage portal protein, pbsx family) to ORF01326 (a site-specific recombinase, phage integrase family). PXO99^A ^contains a second, smaller prophage element spanning six genes from 2366221–2371236.

All 19 of the TAL effector genes show an unusual composition and correspond to peaks in Figure 7. Because the TAL effectors are adjacent to transposases, they too might have originated in another species, possibly as a single-copy gene that later expanded in number in Xoo or a progenitor. Conservation of the unusual composition in all members of the family might also reflect strong functional constraints.

### Hypothetical proteins

A significant fraction of predicted genes in most bacterial genomes are annotated as hypothetical proteins. These open reading frames (ORFs) are predicted computationally, but because they lack sequence homology to other species, they cannot be assigned a name. An unknown number of these predicted genes are likely to be false predictions, and for most genomes there has been little basis for distinguishing true genes at the time of sequencing. For PXO99^A^, we took advantage of the related MAFF and KACC genomes to improve upon the usual set of hypothetical predicted genes. Multiple sequence alignments among several closely-related species often reveal that the ORFs of hypothetical proteins are not maintained in sister species; i.e., they contain in-frame stop codons. Although it is possible that these interrupted ORFs are functional in only one of the species, a more parsimonious explanation is simply that the original gene prediction was wrong. This strategy has been used, for example, to identify several hundred incorrectly annotated genes in *S. cerevisiae *[39], using three related yeast genomes.

We aligned the DNA sequences for all 1273 hypothetical proteins in PXO99^A ^to the corresponding sequences in MAFF, KACC, *X. axonopodis pv. citri*, *X. campestris pv. campestris*, and *X. campestris pv. vesicatoria*. From these alignments, we identified all predicted PXO99^A ^genes with premature stop codons or crippling frameshift mutations in any other species. From these data, we identified 78 ORFs with multiple lines of evidence that they did not represent true genes; these predicted genes were deleted from the annotation.

## Discussion

Nearly 30 distinct bacterial blight resistance genes from different rice varieties and wild relatives have been identified and many have been used in breeding programs for disease control [7], but in several instances, resistance has broken down as new, virulent strains of Xoo have emerged [12,40-42]. Understanding mechanisms that account for the rapid emergence of new pathogen genotypes, and identifying Xoo genes involved in pathogenic adaptation are important goals toward developing durable disease control strategies. The complete genome sequence of strain PXO99^A ^and its comparison to two previously sequenced strains, KACC10331 and MAFF311018, that we have presented here, provide new insights that advance these goals.

Because MAFF and KACC are highly similar in genome content and organization, our comparative analysis focused largely on PXO99^A ^and MAFF. This analysis revealed a remarkable plasticity of the Xoo genome. This plasticity is most strikingly evident in the large number of major rearrangements and indels between these strains. On a smaller scale, differences are prevalent in the inventories of TAL effector genes in PXO99^A ^and MAFF. Also, a number of indels exist that represent genes shared by both strains but present in higher copy in PXO99^A^, including several IS elements. All of these differences suggest that the Xoo genome evolves rapidly. This conclusion is perhaps best supported however by the 212 kb sequence duplication in PXO99^A ^that we discovered using a new and powerful application, the Hawkeye assembly diagnostics tool, and which we confirmed by PCR amplification of the repeat junction. The duplication represents a remarkably recent event, with only a single nucleotide difference differentiating between the two copies in PXO99^A^.

Gene duplication contributes to gene diversification, allowing for unconstrained evolution of otherwise indispensable sequences. The abundance of duplications in PXO99^A ^suggests that they are an important source of genomic variation for Xoo. As made clear by analysis of the 212 kb repeat, IS elements play an important role in generating duplications. And they clearly can generate other types of genome modifications as well, including rearrangements and inversions, and insertions or deletions that can lead to acquisition, modification, or loss of gene content [20]. Indeed, 7 out of 10 of the major rearrangements in the PXO99^A ^genome relative to MAFF are associated with IS elements. The presence of ISXo5 at both ends of the 38.8 kb locus containing the non-fimbrial adhesin-like genes in PXO99^A^, compared with its presence in single copy in place of this locus in MAFF and KACC provides a patent example of an IS mediated genome modification that resulted either in an excision (from the MAFF and KACC lineage), or an integration of DNA (in the PXO99^A ^lineage). Our analysis highlights also an important role for phage as a source of genomic variation for Xoo. The PXO99^A ^sequence revealed numerous differences from MAFF related to phage integration, including the presence of genes that clearly originated in distantly related organisms. Yet another template for genome modification, and a particularly interesting characteristic of the Xoo genomes, are the TAL effector genes. As virulence factors and triggers of host resistance, differences in TAL effector gene content have been associated for some time with phenotypic diversity. Comparison of MAFF and PXO99^A ^provided clear evidence of the involvement of homologous recombination among these genes in generating differences in their structure and copy number at genomic locations that were otherwise conserved, indicating that the sequences themselves play a major role in generating that diversity.

Included among the 19 TAL effector genes in PXO99^A ^are *pthXo1*, a major virulence determinant not present in other strains [29] and *avrXa27*, a cultivar specificity determinant [16]. There is evidence also that the TAL effector gene *pthXo7 *is important in the virulence of PXO99^A ^on plants containing the recessive resistance gene *xa5 *[14,32]. Significantly, *xa5 *is prevalent among the Aus-Boro lines of rice, which originated in Nepal and Bangladesh, the geographical region that likely gave rise to PXO99 [13]. These and other observations firmly establish a role for TAL effector genes in strain-specific adaptation. The differences in TAL effector gene content and structure between the geographically distinct strains PXO99^A ^and MAFF further underscore this role, and the importance of understanding the diversity of TAL effector functions.

The non-fimbrial adhesin-like genes *fhaB*, *fhaB1*, and *fhaX *and the transport gene *fhaC *we discovered at the 38.8 kb locus in PXO99^A ^that is missing in MAFF and KACC are additional intriguing candidates for adaptations to certain host genotypes or environmental conditions. Homologs of *fhaB *and *fhaC *are present in a number of plant and animal pathogenic bacteria [43]. MAFF and KACC encode other non-fimbrial adhesins, which are also present and highly conserved in PXO99^A^. Thus, it seems likely that the *fha *genes are not essential pathogenicity factors in PXO99^A^. However, mutational analysis might reveal a quantitative effect on virulence, or a differential effect in certain rice varieties or under different temperatures. Other proteins encoded at the locus that are of interest from the perspective of host-pathogen interactions include a putative ice nucleation protein and a putative colicin with an associated transporter protein.

Complete genome sequences are available for a number of members of other *Xanthomonas *species, including *X. campestris *pv. campestris, the causal agent of black rot in crucifers, [44,45]*X. axonopodis *pv. citri, which causes citrus canker, and *X. campestris *pv. vesicatoria, which is responsible for bacterial spot in tomato and pepper plants [46]. Whole genome alignments revealed several inversions, indels, and rearrangements in these genomes relative to one another [46]. Thus the genus as a whole shows a high degree of genomic variation. Even in this context however, the differences uncovered here in structure and content of the PXO99^A ^versus the MAFF and KACC genomes are striking. Notably, Xoo strains contain the greatest number and diversity of IS elements of all the sequenced xanthomonads, and the size of the CRISPRs in the strains discussed here suggests a long history of interaction with phage. *X. oryzae *strains are also unusual in their abundance of TAL effector genes. None of the other sequenced *Xanthomonas *strains have more than four TAL effector genes, and some have none. Though a comprehensive survey has not been done, large numbers of TAL effector genes are only known to exist elsewhere in strains of *X. campestris *pv. malvacearum, a pathogen of another ancient and genetically diverse domesticated crop plant, cotton [47], and, curiously, in *Xanthomonas *strains that infect mango [48]. It is tempting to speculate for *X. oryzae *that the diversification of its host through millennia of cultivation around the world favored an amplification of elements in the pathogen that confer genome plasticity and adaptability, including IS elements, phage, and the repeat-dominated TAL effector genes.

It is interesting that in contrast to the East Asian MAFF and KACC strains, the ancestry of PXO99^A ^is likely centered in South Asia [13], one of at least three probable sites of domestication of rice [6]. As described here, PXO99^A ^has a larger genome and a greater number of strain-specific genes than its close relatives MAFF and KACC. This greater size and complexity may be a consequence of this strain having derived from a lineage that evolved near a center of origin for its host, which would be expected to have a greater diversity of host genotypes than other locations.

## Conclusion

The genome sequence of PXO99^A ^and its comparison to those of strains MAFF and KACC provide direct evidence that the Xoo genome is highly plastic and rapidly evolving. Our analysis has revealed sources of genomic variation and identified candidates for strain-specific adaptations of this pathogen. These findings help to explain the extraordinary diversity of Xoo genotypes and races that have been isolated from around the world [9,10,12,13,49-55] and even from within a particular country or region [51,56-60]. Our study also has highlighted particular classes of genes as important targets for functional analysis toward development of better, broader-spectrum and more durable control measures.

## Methods

### Sequencing

Bacterial genomic DNA was randomly sheared by nebulization, end-repaired with consecutive BAL31 nuclease and T4 DNA polymerase treatments, and size-selected using gel electrophoresis on 1% low-melting-point agarose. After ligation to BstXI adapters, DNA was purified by three rounds of gel electrophoresis to remove excess adapters, and the fragments were ligated into the vector pHOS2 (a modified pBR322 vector) linearized with BstXI. The pHOS2 plasmid contains two BstXI cloning sites immediately flanked by sequencing primer binding sites. These features reduce the frequency of non-recombinant clones, and reduce the amount of vector sequences at the end of the reads. Two libraries with average insert size of 4.5 kb and 10 kb were constructed. The ligation reactions were electroporated into *E. coli*. Clones were plated onto large format (16 × 16 cm) diffusion plates prepared by layering 150 ml of fresh antibiotic-free agar onto a previously set 50-ml layer of agar containing antibiotic. Colonies were picked for template preparation, inoculated into 384-well blocks containing liquid media, and incubated overnight with shaking. High-purity plasmid DNA was prepared using the DNA purification robotic workstation custom-built by Thermo CRS (Thermo Fisher Scientific, Inc.) and based on the alkaline lysis miniprep [61] and isopropanol precipitation. DNA precipitate was washed with 70% ethanol, dried, and resuspended in 10 mM Tris HCl buffer containing a trace of blue dextran. The yield of plasmid DNA was approximately 600–800 ng per clone, providing sufficient DNA for at least four sequencing reactions per template. Sequencing was done using di-deoxy sequencing method [62]. Two 384-well cycle-sequencing reaction plates were prepared from each plate of plasmid template DNA for opposite-end, paired-sequence reads. Sequencing reactions were completed using the Big Dye Terminator chemistry and standard M13 forward and reverse primers. Reaction mixtures, thermal cycling profiles, and electrophoresis conditions were optimized to reduce the volume of the Big Dye Terminator mix and to extend read lengths on the AB3730xl sequencers (Applied Biosystems). Sequencing reactions were set up by the Biomek FX pipetting workstations. Robots were used to aliquot and combine templates with reaction mixes consisting of deoxy- and fluorescently labeled dideoxynucleotides, DNA polymerase, sequencing primers, and reaction buffer in a 5 μl volume. After 30–40 consecutive cycles of amplification, reaction products were precipitated by isopropanol, dried at room temperature, resuspended in water, and transferred to an AB3730xl sequencer. 8,700 and 52,100 high-quality reads from the 4.5 kb and 10 kb insert libraries, respectively, were generated with an average trimmed sequence read length of 821 bp and a success rate of 93%. After initial assembly, gaps were closed by primer walking on plasmid templates, sequencing genomic PCR products that spanned the gaps, and by transposon insertion and sequencing of selected 10 kb shotgun clones.

### Assembly and annotation

Multiple rounds of assembly were performed, beginning with the shotgun reads and later including additional finishing reads. In the final assembly, 65,620 reads were trimmed to remove vector and low-quality sequence, and then assembled using Celera Assembler [63]. The large (212 kb) tandem repeat was initially collapsed into one copy, which had twice the depth of coverage of the rest of the genome. This anomaly was detected and corrected to two copies after analysis aided by the Hawkeye assembly diagnosis software [23]. Protein-coding genes were identified using Glimmer 3.0, which includes an algorithm to identify ribosome binding sites for each gene. Transcription terminators were predicted using TransTermHP [66] with parameter settings expected to yield over 90% accuracy. Transfer RNAs were identified with tRNAScanSE [67]. Regions with neither Glimmer predictions nor RNA genes were searched in all six frames using blastx [68] to identify any missed proteins, and all annotations were manually curated as described previously [69], using the Manatee online annotation system [70]. The origin and terminus of replication was determined using GC-skew analysis [18], which indicates an origin near position 50 kb and termini near 2,370 kb or 2,510 kb. The chromosome replication initiator gene dnaA, which is commonly found near the origin, is at position 45. Oligomer skew analysis [71], which identifies 8-mers preferentially located on the leading strand, indicates an origin at 4,895 kb (30 kb from the end of the genome) and a terminus at 2,381 kb, based on multiple 8-mers including CCCTGCCC and AGGACCAT. These 8-mers occur 328/376 and 218/248 times (over 87%) on the leading strand; for CCCTGCCC the likelihood that this occurred by chance is 3.6x10^-45^. To determine genome rearrangements, the MUMmer/Nucmer suite of genome alignment programs [72] was used to align Xoo PXO99^A ^to the MAFF and KACC strains as well as to all other *Xanthomonas *genomes.

### PCR amplification of genes at the non-fimbrial adhesin encoding locus

Genomic DNA was isolated from PXO99^A^, BXO8, Nepal624, KACC10331 and MAFF311018 strains according to the procedure described by Leach et. al. [50]. PCR was performed using a set of gene specific primers listed in Additional file 2.

### Genome data

The PXO99^A ^complete, annotated genome has been deposited in Genbank under accession number CP000967. The traces have been deposited in the NCBI Trace Archive [73] and the complete assembly is in the NCBI Assembly Archive [74].

## Authors' contributions

SLS, JEL, FFW, and AJB conceived the project. SLS, PDR, and AJB coordinated and oversaw the project. SLS and PDR managed all genomic sequencing. DP and MCS did the initial assembly of the genome. DR directed the sequence finishing and gap closure activities. MCS, AMP, and ALD created the final assembly. RM was in charge of the initial, semi-automated genome annotation. MCS, CT, and SLS carried out the overall structural analysis of the genome. PBP and RVS performed the whole genome alignments for phylogenetic analysis. DK, CT, DDS, and SLS compared the gene content of PXO99^A ^and MAFF. CT and MAVS analyzed IS elements. GA and RVS analyzed the adhesin locus. MCS, ALD, and SLS discovered and characterized the 212 kb duplication. FFW carried out the TAL effector analysis, assisted by RK and AJB. CT documented rearrangements in the PXO99^A ^genome relative to MAFF. DDS, SLS and RK investigated the CRISPRs. SeT, AF, and HO validated the MAFF assembly. SLS identified regions of possible lateral gene transfer. DK optimized annotation of hypothetical protein genes. SeT, AF, GA, GJ, AP, PBP, RVS, HI, DFM, BS, VV, JMD, RPR, HH, ShT, SWL, PCR, RVS, MAVS, JEL, FFW, and AJB contributed to the manual annotation. SLS and AJB drafted the manuscript, assisted by PDR, SeT, GA, PBP, RVS, RK, MAVS, JEL, and FFW. All authors approved the final manuscript.

## Supplementary Material

Additional file 1 **Supplementary Figure 1**. Phylogenetic relationships among *X. oryzae *pv. oryzae (Xoo) strains PXO99^A^, KACC10331, and MAFF311018, and *X. oryzae *pv. oryzicola (Xoc) strain BLS256 based on whole genome alignment. Click here for file

Additional file 2 **Supplementary Tables**. Supplementary Table 1, Genes unique to PXO99^A ^and unique to MAFF311018; Supplementary Table 2, Primers used to amplify genes at the non-fimbrial adhesin encoding locus; Supplementary Table 3, Primers used to confirm the 212 kb direct repeat. Click here for file
