# Genome sequence and rapid evolution of the rice pathogen *Xanthomonas oryzae *pv. oryzae PXO99^A^

**DOI:** 10.1186/1471-2164-9-534

**Published:** 2008-11-11

**Authors:** Steven L Salzberg, Daniel D Sommer, Michael C Schatz, Adam M Phillippy, Pablo D Rabinowicz, Seiji Tsuge, Ayako Furutani, Hirokazu Ochiai, Arthur L Delcher, David Kelley, Ramana Madupu, Daniela Puiu, Diana Radune, Martin Shumway, Cole Trapnell, Gudlur Aparna, Gopaljee Jha, Alok Pandey, Prabhu B Patil, Hiromichi Ishihara, Damien F Meyer, Boris Szurek, Valerie Verdier, Ralf Koebnik, J Maxwell Dow, Robert P Ryan, Hisae Hirata, Shinji Tsuyumu, Sang Won Lee, Young-Su Seo, Malinee Sriariyanum, Pamela C Ronald, Ramesh V Sonti, Marie-Anne Van Sluys, Jan E Leach, Frank F White, Adam J Bogdanove

**Affiliations:** 1 Center for Bioinformatics and Computational Biology, University of Maryland, College Park, MD 20742, USA; 2 The Institute for Genomic Research, Rockville, MD 20850, USA; 3 Institute for Genome Sciences, University of Maryland, Baltimore, MD 21201, USA; 4 Laboratory of Plant Pathology, Kyoto Prefectural University, Sakyo, Kyoto 606-8522, Japan; 5 Department of Genetic Resources, National Institute of Agrobiological Sciences, Kannondai, Tsukuba 305-8602, Japan; 6 Current address: J. Craig Venter Institute, Rockville, MD 20850, USA; 7 Current address: National Center for Biotechnology Information, National Institutes of Health, Bethesda, MD 20894, USA; 8 Centre for Cellular and Molecular Biology, Council of Scientific and Industrial Research, Hyderabad, India; 9 Institute of Himalayan Bioresource Technology, Council of Scientific and Industrial Research, Palampur, India; 10 Department of Bioagricultural Sciences and Pest Management, Colorado State University, Fort Collins, CO, USA; 11 Department of Plant Pathology, Iowa State University, Ames, IA, USA; 12 Institut de la Recherche pour le Developpement, 911 Av. Agropolis, Montpellier, 34090, France; 13 BIOMERIT Research Centre, BioSciences Institute, University College Cork, Cork, Ireland; 14 Graduate School of Natural Science & Technology, Shizuoka University, 836 Ohya, Suruga-ku, Shizuoka, 422-8017, Japan; 15 Department of Plant Pathology, UC Davis, Davis, CA 95616, USA; 16 Departamento de Botânica, IB-USP, Sao Paulo, SP, Brazil; 17 Department of Plant Pathology, Kansas State University, Manhattan, KS, USA

## Correction

Following the publication of the article 'Genome sequence and rapid evolution of the rice pathogen *Xanthomonas oryzae *pv. oryzae PXO99^A^. BMC Genomics 2008, 9:204' [1], the submitting author became aware that two co-authors had been omitted. Therefore, this article, which states the contributions that these authors made to the original article, has been submitted as a correction. We apologize for any inconvenience this oversight may have caused.

## Corrected author list

Steven L Salzberg, Daniel D Sommer, Michael C Schatz, Adam M Phillippy, Pablo D Rabinowicz, Seiji Tsuge, Ayako Furutani, Hirokazu Ochiai, Arthur L Delcher, David Kelley, Ramana Madupu, Daniela Puiu, Diana Radune, Martin Shumway, Cole Trapnell, Gudlur Aparna, Gopaljee Jha, Alok Pandey, Prabhu B Patil, Hiromichi Ishihara, Damien F Meyer, Boris Szurek, Valerie Verdier, Ralf Koebnik, J Maxwell Dow, Robert P Ryan, Hisae Hirata, Shinji Tsuyumu, Sang Won Lee, Young-Su Seo, Malinee Sriariyanum, Pamela C Ronald, Ramesh V Sonti, Marie-Anne Van Sluys, Jan E Leach, Frank F White and Adam J Bogdanove.

## Corrected authors' contributions

SLS, JEL, FFW, and AJB conceived the project. SLS, PDR, and AJB coordinated and oversaw the project. SLS and PDR managed all genomic sequencing. DP and MCS did the initial assembly of the genome. DR directed the sequence finishing and gap closure activities. MCS, AMP, and ALD created the final assembly. RM was in charge of the initial, semi-automated genome annotation. MCS, CT, and SLS carried out the overall structural analysis of the genome. PBP and RVS performed the whole genome alignments for phylogenetic analysis. DK, CT, DDS, and SLS compared the gene content of PXO99^A ^and MAFF. CT and MVS analyzed IS elements. GA and RVS analyzed the adhesin locus. MCS, ALD, and SLS discovered and characterized the 212 kb duplication. FFW carried out the TAL effector analysis, assisted by RK and AJB. CT documented rearrangements in the PXO99^A ^genome relative to MAFF. DDS, SLS and RK investigated the CRISPRs. SeT, AF, and HO validated the MAFF assembly. SLS identified regions of possible lateral gene transfer. DK optimized annotation of hypothetical protein genes. SeT, AF, GA, GJ, AP, PBP, RVS, HI, DFM, BS, VV, JMD, RPR, HH, ShT, SWL, YS, MS, PCR, RVS, MVS, JEL, FFW, and AJB contributed to the manual annotation. SLS and AJB drafted the manuscript, assisted by PDR, SeT, GA, PBP, RVS, RK, MVS, JEL, and FFW. All authors approved the final manuscript.
