# Supplementary material for: Genome sequence and rapid evolution of the rice pathogen Xanthomonas oryzae pv. oryzae PXO99A
Source: BMC Genomics. 2008 May 1;9:204. doi: 10.1186/1471-2164-9-204 (PMC2432079; doi:10.1186/1471-2164-9-204)
Supplement: Additional file 1 — Supplementary Figure 1. Phylogenetic relationships among X. oryzae pv. oryzae (Xoo) strains PXO99A, KACC10331, and MAFF311018, and X. oryzae pv. oryzicola (Xoc) strain BLS256 based on whole genome alignment. [file 1471-2164-9-204-S1.pdf]

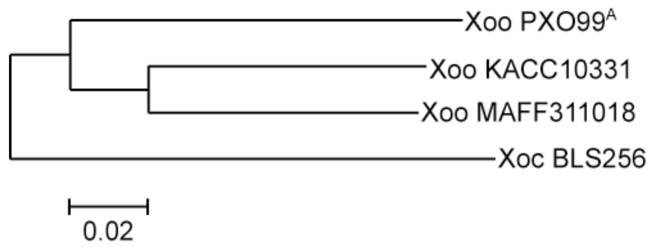

Supplementary Figure 1. Phylogenetic relationships among *X. oryzae* pv. oryzae (Xoo) strains PXO99^{A}, KACC10331, and MAFF311018, and *X. oryzae* pv. oryzicola (Xoc) strain BLS256 based on whole genome alignment generated using MAUVE 2.1.1. The tree is rooted to BLS256.
