# Supplementary material for: Genome sequence and rapid evolution of the rice pathogen Xanthomonas oryzae pv. oryzae PXO99A
Source: BMC Genomics. 2008 May 1;9:204. doi: 10.1186/1471-2164-9-204 (PMC2432079; doi:10.1186/1471-2164-9-204)
Supplement: Additional file 2 — Supplementary Tables. Supplementary Table 1, Genes unique to PXO99A and unique to MAFF311018; Supplementary Table 2, Primers used to amplify genes at the non-fimbrial adhesin encoding locus; Supplementary Table 3, Primers used to confirm the 212 kb direct repeat. [file 1471-2164-9-204-S2.pdf]

Supplementary Table 1A: Genes present in Xoo PXO99[A] and missing from Xoo MAFF311018.

Gene coordinates	Length	ID	Name	Description
34958..35620	663	ORF03448	-	conserved hypothetical protein
35774..36568	795	ORF03447	lasA	LasA
36546..36662	117	ORF03446	-	hypothetical protein
36724..37209	486	ORF03445	-	conserved hypothetical protein
37568..38212	645	ORF03444	-	proline-betaine transporter
38213..38788	576	ORF03443	-	proline/betaine transporter
139333..139536	204	ORF03345	-	conserved hypothetical protein
139600..139743	144	ORF05422	-	hypothetical protein
197682..197795	114	ORF03662	-	hypothetical protein
1013314..1013622	309	ORF04418	-	conserved hypothetical protein
1180249..1180515	267	ORF04601	-	two-component system sensor protein
1212055..1213419	1365	ORF04562	-	extracellular protease
1213532..1213648	117	ORF05505	-	hypothetical protein
1392096..1393283	1188	ORF04735	pliMCI	DNA (cytosine-5)-methyltransferase PliMCI
1394831..1395505	675	ORF04733	-	conserved hypothetical protein
1395498..1396916	1419	ORF04731	tnpA	transposase TnpA, ISL3 family
1502716..1502835	120	ORF05529	-	hypothetical protein
1557643..1557897	255	ORF00292	-	hypothetical protein
1562602..1562886	285	ORF00288	-	transposase
1577774..1577902	129	ORF00276	-	conserved hypothetical protein
1875626..1875754	129	ORF00499	-	conserved hypothetical protein
2282450..2282563	114	ORF00642	-	hypothetical protein
2282620..2283132	513	ORF00641	-	phosphinothricin N-acetyltransferase
2286236..2286640	405	ORF00637	-	conserved hypothetical protein
2287361..2288338	978	ORF00636	-	ATPase, AAA family
2288335..2290563	2229	ORF00635	-	peptidase S8 and S53, subtilisin, kexin, sedolisin
2293644..2294663	1020	ORF00631	-	cointegrate resolution protein T
2294920..2296527	1608	ORF00630	-	conserved hypothetical protein

2296674..2297741	1068	ORF00629	-	conserved hypothetical protein
2297807..2298085	279	ORF05830	arsR	transcriptional regulator, ArsR family
2298082..2298612	531	ORF00628	-	arsenate reductase
2298623..2299036	414	ORF00627	-	arsenate reductase
2299033..2299758	726	ORF00626	arsH	arsenical resistance protein ArsH
2299777..2301081	1305	ORF00625	-	arsenical membrane pump
2301719..2302687	969	ORF00624	-	cointegrase
2302697..2305657	2961	ORF00623	-	transposase
2374988..2375713	726	ORF00555	-	hypothetical protein
2375676..2377568	1893	ORF00554	-	hypothetical protein
2377696..2378598	903	ORF00553	-	hypothetical protein
2379714..2379845	132	ORF00552	-	hypothetical protein
2667950..2668135	186	ORF00946	-	hypothetical protein
2880037..2880222	186	ORF06220	-	hypothetical protein
3265953..3266066	114	ORF01634	-	hypothetical protein
3266814..3266927	114	ORF01632	-	hypothetical protein
3382894..3383220	327	ORF01532	-	conserved hypothetical protein
3537831..3539210	1380	ORF01367	-	conserved hypothetical protein
3557949..3558251	303	ORF01342	-	phage tail protein E
3561289..3561690	402	ORF01339	-	gpU
3561687..3562673	987	ORF01338	-	bacteriophage P2 gpD protein
3563281..3563712	432	ORF01337	-	phage-related protei
3563987..3564130	144	ORF01336	-	hypothetical protein
3564228..3564365	138	ORF05676	-	hypothetical protein
3564459..3564656	198	ORF01335	-	N-acetylglucosamine-6-phosphate deacetylase
3564653..3564865	213	ORF06037	-	conserved hypothetical protein
3567882..3568100	219	ORF01333	-	hypothetical protein
3568398..3568637	240	ORF01331	-	conserved hypothetical protein
3569014..3569136	123	ORF01330	-	conserved hypothetical protein
3569150..3569311	162	ORF01329	-	conserved hypothetical protein
3569367..3569576	210	ORF01328	-	conserved hypothetical protein
3569573..3569797	225	ORF01327	-	conserved hypothetical protein
3570700..3571728	1029	ORF01326	-	site-specific recombinase,

				phage integrase family
3827147..3828025	879	ORF02076	-	conserved hypothetical protein
3829018..3829473	456	ORF02074	-	HsdS polypeptide, part of CfrA family
3830403..3831872	1470	ORF02073	-	type I restriction enzyme EcoEI M protein
3833299..3835680	2382	ORF02070	-	type I restriction enzyme EcoAI R protein
3873652..3876546	2895	ORF02039	-	EF hand domain protein
3878818..3881073	2256	ORF02037	-	conserved hypothetical protein
4762324..4762572	249	ORF03024	-	methyltransferase
4788763..4788957	195	ORF02994	-	Rhs family protein
4790407..4792539	2133	ORF02991	FhaB1	filamentous haemagglutinin, N-terminal:Adhesin HecA 20-residue repeat x2
4793973..4794086	114	ORF05768	-	hypothetical protein
4797040..4797390	351	ORF06047	-	radical SAM domain protein
4798830..4799168	339	ORF02988	-	hypothetical gene
4800219..4801496	1278	ORF02987	-	putative secretion protein
4801493..4803496	2004	ORF02986	-	ABC transporter, ATP-binding protein
4803861..4804559	699	ORF02985	-	TPR repeat [Prochlorococcus marinus str. NATL2A]
4805791..4806084	294	ORF02982	-	transposase IS3
4806129..4806941	813	ORF02981	-	putative transposase
4806979..4807125	147	ORF05769	-	ISXoo9 transposase orfB [Xanthomonas oryzae pv. oryzae MAFF 311018]
4807476..4807631	156	ORF02979	-	filamentous haemagglutinin
4809387..4811960	2574	ORF02977	fhaX	filamentous haemagglutinin
4812238..4812369	132	ORF05770	-	hypothetical protein
4812409..4822989	10581	ORF02976	fhaB	filamentous haemagglutinin; haemagglutination activity domain protein
4823155..4824867	1713	ORF02975	fhaC	outer membrane hemolysin activator protein
4826699..4827529	831	ORF02973	-	ice nucleation protein
4905246..4905359	114	ORF02895	-	hypothetical protein
5215279..5216559	1281	ORF03510	-	prophage Lp2 protein 6

Supplementary Table 1B: Genes found in Xoo MAFF and missing from Xoo PXO99[A]

Gene coordinates	Length	ID	Name	Description

483297..484136	279	XOO0441	-	hypothetical protein
550463..551077	204	XOO0504	-	hypothetical protein
551077..552312	411	XOO0505	-	hypothetical protein
634634..634798	54	XOO0584	-	hypothetical protein
634883..635569	228	XOO0585	-	putative Zn-dependent alcohol dehydrogenase
1072351..1072989	212	XOO0982	-	hypothetical protein
1074162..1074581	139	XOO0984	-	hypothetical protein
1074578..1074793	71	XOO0985	-	hypothetical protein
1076373..1076708	111	XOO0987	-	hypothetical protein
1461046..1461996	316	XOO1339	-	hypothetical protein
1463190..1463681	163	XOO1341	-	hypothetical protein
1466111..1467058	315	XOO1343	-	hypothetical protein
1467051..1467401	116	XOO1344	-	hypothetical protein
1468154..1468600	148	XOO1345	-	hypothetical protein
1469697..1470368	223	XOO1348	-	hypothetical protein
1470418..1472526	702	XOO1349	-	hypothetical protein
1619087..1619383	98	XOO1478	-	hypothetical protein
1712101..1712505	134	XOO1556	-	hypothetical protein
1712511..1712660	49	XOO1557	-	hypothetical protein
1712950..1713660	236	XOO1558	-	pseudouridylate synthase
1713895..1714101	68	XOO1559	-	hypothetical protein
1714305..1714733	142	XOO1560	-	hypothetical protein
1714737..1715297	186	XOO1561	-	hypothetical protein
1715895..1716467	190	XOO1562	-	membrane transport protein
1723804..1724988	394	XOO1570	-	phage-related integrase
1724988..1725248	86	XOO1571	-	hypothetical protein
1725206..1725412	68	XOO1572	-	hypothetical protein
1725409..1725681	90	XOO1573	-	hypothetical protein
1725920..1726195	91	XOO1574	-	hypothetical protein
1726188..1726343	51	XOO1575	-	hypothetical protein
1726357..1726767	136	XOO1576	-	hypothetical protein
1727268..1727486	72	XOO1578	-	hypothetical protein
1730501..1730713	70	XOO1580	-	hypothetical protein
1730710..1730988	92	XOO1581	-	hypothetical protein
1730999..1731319	106	XOO1582	-	hypothetical protein
1731651..1732088	145	XOO1583	-	hypothetical protein
1732749..1733735	328	XOO1584	-	phage-related tail protein
1733732..1734133	133	XOO1585	-	phage-related tail protein
1737171..1737473	100	XOO1588	-	phage-related tail protein
1759264..1760550	428	XOO1617	-	polymerase V subunit
1760778..1761113	111	XOO1618	-	hypothetical protein
1836657..1837775	372	XOO1678	-	hypothetical protein
1837772..1838443	223	XOO1679	-	hypothetical protein
1970102..1970752	216	XOO1785	-	hypothetical protein
2170619..2170966	115	XOO1964	-	hypothetical protein

2370591..2371238	215	XOO2140	-	TrbP protein
2371240..2372427	395	XOO2141	-	hypothetical protein
2372427..2372756	109	XOO2142	-	hypothetical protein
2372756..2374204	482	XOO2143	-	hypothetical protein
2374299..2374529	76	XOO2144	-	hypothetical protein
2374748..2375044	98	XOO2145	-	V protein
2375041..2376081	346	XOO2146	-	replication initiation protein
2376234..2376446	70	XOO2147	-	hypothetical protein
2379597..2379788	63	XOO2153	-	hypothetical protein
2380021..2380488	155	XOO2154	-	hypothetical protein
2380402..2380914	170	XOO2155	-	hypothetical protein
2448781..2449662	293	XOO2210	-	hypothetical protein
2987332..2988084	250	XOO2659	-	hypothetical protein
3007526..3007942	138	XOO2674	-	hypothetical protein
3007939..3008190	83	XOO2675	-	hypothetical protein
3008628..3009263	211	XOO2676	-	hypothetical protein
3011170..3011778	202	XOO2678	-	hypothetical protein
3011775..3012089	104	XOO2679	-	hypothetical protein
3695045..3698224	1059	XOO3254	-	type I restriction-modification system endonuclease
3698224..3698898	224	XOO3255	-	hypothetical protein
3698898..3699644	248	XOO3256	-	hypothetical protein
3699641..3700174	177	XOO3257	-	hypothetical protein
3702759..3703472	237	XOO3260	-	hypothetical protein
3703465..3703899	144	XOO3261	-	nucleotidyltransferase
3703910..3705724	604	XOO3262	-	type I restriction system adenine methylase
3705742..3706734	330	XOO3263	-	hypothetical protein
3706787..3707095	102	XOO3264	-	hypothetical protein
3769562..3770884	440	XOO3308	-	hypothetical protein
3771391..3771870	159	XOO3309	-	hypothetical protein
3771894..3772358	154	XOO3310	-	hypothetical protein
3772675..3773154	159	XOO3311	-	hypothetical protein
3773178..3773642	154	XOO3312	-	hypothetical protein
3773639..3774535	298	XOO3313	-	hypothetical protein
4214732..4215826	364	XOO3728	-	restriction endonuclease homolog R.XphI
4215823..4217613	596	XOO3729	-	methyltransferase homolog M.XphI

Supplementary table 2: Primers used to amplify genes at the non-fimbrial adhesin encoding locus. The primers were designed based on $PXO99^A$ genome sequence. DSP: Dual Specificity Protein; Fha: Filamentous hemagglutinin; DBP: DNA Binding Protein.

Primers used in the study	Primer sequence
DSPF	5′ GGGGCCGTTTTTCTTCCTCAGCTA 3′

DSPR	5′ GAAGCCCAATAACACCGCGAACAA 3′
FhaCF	5′ CGCCGCGTGGTGTGCTGTGCTTAT 3′
FhaCR	5′ CCGGCATCGTCCAGGCTCAGGGTG 3′
FhaBF	5′ ATGCTCTGCGCGCTCGGCCTTGTC 3′
FhaBR	5′ GCGCGAACGTGGGTGCCTGGCC 3′
FhaXF	5′ CCTGAGCGGCGACACGGTACACAT 3′
FhaXR	5′ CCGTGCTTTCCTCATGCGTGGC 3′
DBPF	5′ CGTCTTGTCGCCGCAGGAATACC 3′
DBPR	5′ GCCGTCCAGGTCGCGCAGATAAC 3′

Supplementary table 3: Primers used to confirm the 212kb direct repeat. The left and right primers were designed outside but close to the central repeat region, and 1073 bp repeat linking the two 212kb direct repeats.

Primer identifier	Primer sequence
T679LEFT2	5′ TTGGGGATTCGTGATTGGAGATGG 3′
T679RIGHT	5′ AGAACCTGTTCACGATCTCCTGAGC 3′
